# Administration of hydro-alcoholic extract of spinach improves oxidative stress and inflammation in high-fat diet-induced NAFLD rats

**DOI:** 10.1186/s12906-021-03396-x

**Published:** 2021-09-03

**Authors:** Ali Amirinejad, Ali Saneei Totmaj, Farzaneh Mardali, Azita Hekmatdoost, Hadi Emamat, Majid Safa, Farzad Shidfar

**Affiliations:** 1 Department of Nutrition, School of Public Health, Iran University of Medical Sciences, Tehran, 1449614535 Iran; 2 Department of Clinical Nutrition and Dietetics, Faculty of Nutrition and Food Technology, National Nutrition and Food Technology, Research Institute, Shahid Beheshti University of Medical Sciences, Tehran, Iran; 3 Student Research Committee, Department of Clinical Nutrition and Dietetics, Faculty of Nutrition and Food Technology, National Nutrition and Food Technology Research Institute, Shahid Beheshti University of Medical Sciences, Tehran, Iran; 4 Department of Hematology and Blood Transfusion, School of Allied Medicine, Iran University of Medical Sciences, Tehran, Iran

**Keywords:** Spinach, NAFLD, NASH, Inflammation, Oxidative stress

## Abstract

**Background:**

Nonalcoholic fatty liver disease (NAFLD) is the most common liver disease worldwide. The aim of this study was to evaluate the effects of hydro-alcoholic extract of spinach (HES) on hepatic and serum measurements of NAFLD in a rat model.

**Methods:**

In the prevention phase, 18 Sprague–Dawley rats were fed a high-fat diet, a high-fat diet plus 400 mg/kg HES, or a chow diet for seven weeks. For the treatment phase, after the induction of NAFLD, they were fed a high-fat diet, a high-fat diet plus 400 mg/kg HES, a chow diet, or a chow diet plus 400 mg/kg HES for four weeks (*n* = 6).

**Results:**

Administration of HES combined with high-fat diet in rats was associated with decreased food intake (*P* < 0.01), weight loss (*P* = 0.01), and increased superoxide dismutase (SOD) (*P* = 0.02) enzyme activity in the liver, at the end of the prevention phase. hs-CRP (*P* < 0.05), PTX-3 (*P* < 0.05), and TNF-α (*P* < 0.05) gene expression in the liver were decreased and PPAR-γ (*P* < 0.05) gene expression in the liver was increased by spinach intake, both in the prevention and treatment phases. Furthermore, administration of spinach in the treatment phase increased serum TAC (*P* = 0.03) and hepatic GPX (*P* = 0.01) enzyme activity.

**Conclusion:**

Taking into account the potential beneficial effects of HES on prevention and treatment of NAFLD in the present study, to confirm these findings, we propose that further clinical trials be conducted on human subjects with NAFLD.

**Supplementary Information:**

The online version contains supplementary material available at 10.1186/s12906-021-03396-x.

## Introduction

Nonalcoholic fatty liver disease (NAFLD) is the most common liver disease worldwide [1]. NAFLD is generally defined as the hepatic accumulation of triglycerides (steatosis) which affects at least 5% of the liver volume or weight, in the absence of consuming significant amounts of alcohol (less than 30 g/day for men and less than 20 g/day for women) as well as other causes of steatosis, such as exposure to certain drugs or toxins [2, 3]. Nonalcoholic steatohepatitis (NASH) is an advanced form of NAFLD characterized by inflammation, apoptosis, and ballooning degeneration [4]. NAFLD is a multifactorial disease caused by different genetic and lifestyle variables. Obesity, insulin resistance, diabetes, hyperlipidemia, and metabolic syndrome are the main risk factors for NAFLD [5, 6]. Lifestyle modifications, including dietary interventions and physical activity, are the major components of medical management in NAFLD [7, 8]. Bariatric surgery is an alternative method for those who fail to attain a significant weight loss [9]. Additionally, medications counteracting insulin resistance, lipid-lowering, and anti-blood pressure agents have been already used in the management of NAFLD [10].

Spinach (*Spinacia Oleracea* L.) is a leafy green vegetable belonging to the Amaranthaceae family. Numerous bioactive ingredients, such as β-carotene, flavonoids, phenolic compounds, lutein, lycopene, linolenic acid, and thylakoids are present in spinach [11, 12]. These phytochemicals have been postulated to be responsible for hypoglycemic, hypolipidemic, anti-obesity, antioxidant, and anti-inflammatory properties of spinach [13]. Thylakoids, major constituents of the chloroplast membranes, delay fat digestion by inhibiting pancreatic lipase/colipase [14, 15], leading to an increase in circulating levels of satiety hormones, including cholecystokinin (CCK). Elevated serum levels of CCK after a thylakoid-rich meal have been reported in several studies [16–19]. In one study, thylakoid intake decreased fat accumulation in the liver by increasing the gene expression of the peroxisome proliferator-activated receptor-gamma (PPAR-γ**)** in the adipose tissue [20].

Among extracellular matrix proteins, increased gene expression and plasma levels of matrix metalloproteinase-9 (MMP-9) have been observed in alcoholic patients, individuals with viral cirrhosis, and especially people with NASH [21, 22]. A pro-inflammatory profile, including high levels of high-sensitivity C-reactive protein (hs-CRP), pentraxin-3 (PTX-3), and tumor necrosis factor-alpha (TNF-α), has been observed in people with NAFLD [23–25]. On the other hand, interleukin-10 (IL-10), a potent anti-inflammatory cytokine, has been shown to be inversely associated with hepatic inflammation [26, 27].

Consumption of spinach decreased liver steatosis and improved glucose and lipid profile in previous animal-based NAFLD models [28, 29]. To further establish the existing data, this study aimed to investigate the effect of supplementation with hydro-alcoholic extract of spinach (HES) on serum levels and hepatic gene expression of some inflammatory and oxidative factors in a rat model of NAFLD.

## Materials and methods

### Preparation of high-fat diet and spinach extract

The high-fat, high-sugar diet was prepared by mixing butter, chow (Behparvar, Tehran, Iran) sugar, and egg; using the method previously established by Emamat et al. [30]. The food was given every other day at the beginning of the dark phase, and the remains were measured and removed after 48 h.

Spinach (*S. oleracea*) was collected from a private farm in Tehran province with the permission from the land owner. A plant specimen identified and kept at the herbarium of the Institute of Medicinal Plants (ACECR) Karaj, Iran with herbarium number 1325(MPIH).

The leaves of spinach were washed and dehydrated under shaded circumstances, and then they were ground to obtain a dried powder. For preparing HES, this powder was immersed in a mixture of methanol/water (70:30, v/v) for 24 h and it was then filtered. This procedure was replicated two more times and then the extract was concentrated in a rotary evaporator at 50 °C. The dried extract was stored in the freezer for distant use. The extraction yield of HES from leaves was 10.4% w/w.

### Animals and experimental method

Forty-two male Sprague–Dawley rats aged 8 weeks (weighing 150 ± 10 g) were purchased from Pasteur Institute (Karaj, Iran) and were housed separately in polycarbonate wire cages in a standard environment (20–22 °C, 50% humidity and 12-h light/dark cycles). After one-week acclimation, the prevention phase was designed for seven weeks and 18 rats were randomly assigned into three groups (*n* = 6 in each group): the first group (PC) were fed a standard chow diet with 1 mL normal saline administered by gavage, the second group (PF) were fed a high-fat diet with 1 mL normal saline by gavage, and the third group (PFS) received a high-fat diet with 400 mg/kg HES (1 mL) by gavage for seven weeks.

In the treatment phase, 24 rats were fed the HF diet for seven weeks in order to induce NAFLD [30], and then they were randomly divided into four groups(*n* = 6 in each group):The first group (TC) were fed a standard chow diet with 1 mL normal saline by gavage, the second group (TCS) were fed a standard chow diet with 400 mg/kg HES (ml) by gavage, the third group received a HF diet with 1 mL normal saline by gavage (TF), and finally the fourth group (TFS) received a HF diet with 400 mg/kg HES (1 mL) by gavage for 4 weeks. Random numbers were generated using online random number generators. The dosage of 400 mg/kg was based on the two studies by Panda et al. which reported the benefits of 14 and 45 days supplementation with HES (400 mg/kg) [31, 32].

Body weights and food intakes were registered as grams every two weeks and twice a week, respectively. At the end of the intervention, the rats were terminated by cardiac puncture under anesthesia using ketamine and xylazine. The animals had free access to food and water in all stages.

All animal procedures were carried out in accordance with the Iran University of Medicals Sciences ethics committee (IR.IUMS.REC) and the study protocol was approved by IR.IUMS.REC with ethics code of IR.IUMS.REC 1396.9511468001.

### Blood and tissue preparation

5 ml blood was obtained by cardiac puncture and centrifuged (2000 g, 10 min, 4 °C) for the clot to be removed. The supernatants were collected for biochemical analysis.

Two samples were obtained from each liver. 300 mg tissue was excised from liver and homogenized in 3 mL phosphate-buffered saline (PBS = 100 mM, pH 7.4). After centrifuging (2000 g, 20 min, 4 °C), the supernatants were collected and stored at − 80 °C for measuring antioxidant enzymes activity. Another sample (70 mg) was incised and preserved in RNA for consequent gene expression assessments.

### Biochemical measurements

Serum total antioxidant capacity (TAC) was measured using colorimetry at the wavelength of 460–490 nm, based on the oxidation-reduction interactions in the sample using a commercial kit (ZellBio GmbH, Germany). Serum Matrix Metalloproteinase-9 (MMP-9) (Sensitivity: 0.01 ng/mL), hs-CRP (sensitivity: 0.09 ng/ml), Pentraxin-3 (PTX3) (Sensitivity: 0.05 ng/mL), and Cholecystokinin (CCK) (Sensitivity: 5 pg/mL) were measured by the means of the biotin double antibody sandwich technology with rat commercial enzyme-linked immunosorbent assay (ELISA) kits (ZellBio GmbH, Germany). The micro ELISA plate provided in this kit had been pre-coated with an antibody specific to the variable. Standards or samples were added to the appropriate micro ELISA plate wells and interacted with the specific antibody. Then a biotinylated detection antibody, specific for the desired variable, and Avidin-Horseradish Peroxidase (HRP) conjugate were successively added to each micro plate well and were incubated. Free components were washed away. The substrate solution was added to each well. Only in those wells that contained the desired variable, biotinylated detection antibody and Avidin-HRP conjugate turned blue in color. The enzyme-substrate reaction was terminated by the addition of a sulphuric acid solution, making the wells turn yellow. The optical density (OD) was measured spectrophotometrically at a wavelength of 450 nm ± 2 nm. The OD value was then considered proportional to the concentration of the desired variable. The concentration of variable in the samples was calculated by comparing the OD of the samples to the standard curve [33, 34].

Liver glutathione peroxidase (GPX) (sensitivity: 5 U/mL) and superoxide dismutase (SOD) (sensitivity: 1 U/mL) activity were measured by colorimetry method (420 nm) using commercial kits (ZellBio GmbH, Germany).

### RNA extraction and real-time polymerase chain reaction analysis

Total RNA was extracted using YTzol pure RNA reagent (Yekta Tajhiz Azma, Tehran, Iran) following the manufacturer’s protocol; the purity was checked by the ratio of absorbance at 260 nm and 280 nm, using Thermo Scientific™ NanoDrop™ One Microvolume UV-Vis Spectrophotometer. cDNA synthesis kit (Yekta Tajhiz Azma, Tehran, Iran), which used random hexamer primer, was employed to synthesize complementary DNA from 2 μg total RNA.

The quantitative RT-PCR was performed using Roche LightCycler® 96 Real-Time PCR System and YTA SYBR Green qPCR Master Mix 2X (Yekta Tajhiz Azma, Tehran, Iran). PCR reaction system contained 1 μL cDNA, 1 μL of the proper forward and reverse primers, and 7.5 μL SYBR Green PCR Master yielding a total volume of 15 mL. The thermal cycling conditions were: 95 °C for 3 min (initial denaturation); 95 °C for 10 s repeated in 40 cycles (denaturation), and 60 °C for 1 min (annealing/extension). The relative expression of target genes were normalized to expression of GAPDH and fold change was calculated using 2^−ΔΔCt^ method [35]. The specificity of the PCR products was verified by melt curve analysis. The sequences of primers used in qPCR are presented in Table 1.

**Table 1:** The sequence of primers used for gene expression

Gene Name	Sequences	Accession No.	No. of first and last Nt.	Product length
GAPDH-FW	5′-CCTGTGACTTCAACAGCAACTC-3´	NM_017008.4	917–938	177 bp
GAPDH-RW	5′-GGTGGTCCAGGGTTTCTTACTC-3´		1072–1093	
PPAR-γ-FW	5′-GTTCACAAGAGCTGACCCAATG-3´	NM_013124.3	332–353	135 bp
PPAR-γ-RW	5′-TGTGGCCTGTTGTAGAGTTGG-3´		446–466	
TNF-α-FW	5′-CCAAATGGGCTCCCTCTCATC-3´	NM_012675.3	337–357	112 bp
TNF-α-RW	5′-CCGCTTGGTGGTTTGCTAC-3´		430–448	
IL-10-FW	5′-GCGACGCTGTCATCGATTTC-3´	NM_012854.2	370–389	153 bp
IL-10-RW	5′-AGTGTCACGTAGGCTTCTATG C-3´		501–522	

### Statistical analysis

All results are expressed as mean ± SD. The analysis was performed using the SPSS v.22 software (Chicago, IL, USA). The normality of data was analyzed by the means of the Kolmogorov–Smirnov test. Regarding the data with a normal distribution, statistical disparities between groups were assessed using the one-way ANOVA test, followed by the LSD post-hoc test; the Kruskal-Wallis test was used in the case of non-normality. *P* < 0.05 was considered statistically significant for all the tests mentioned.

## Results

### Prevention phase

All of the 18 samples were used for biochemical analysis and the quantification of gene expression in the prevention phase (*n* = 6 in each group). Total weight gain and food intake, serum levels of inflammation, oxidative stress biomarkers, and antioxidant activity in the liver are presented in the Table 2. Total weight gain and food intake were significantly greater in PF group compared to PC and PFS groups (*P* < 0.01). Additionally, serum levels of hs-CRP and PTX-3 in the PF group were significantly greater than those of PFS group after seven weeks of intervention (*P =* 0.01). The liver activity of SOD was also higher in the PFS group compared to the PF group (*P =* 0.02). No significant differences were observed in serum MMP-9, CCK, TAC, and liver activity of GPX among groups.

**Table 2:** Weight gain, total food intake, serum inflammation and oxidative stress biomarkers, and antioxidant enzyme activity in liver at the end of the prevention phase

Variables	Control (***n*** = 6)	High-fat (***n*** = 6)	High-fat + Spinach (***n*** = 6)	***P*** value, ANOVA
**Weight gain (g)**	94 ± 19.1^a^	142.8 ± 26^b^	116.1 ± 16.2^a^	< 0.01^*^
**Food Intake (g)**	152.4 ± 13.9^a^	175.8 ± 12.3^b^	159.9 ± 8.7^a^	0.01^*^
**CCK (ng/l)**	139.1 (103–187.5)	155.6 (128.5–216.5)	124 (114.9–133.1)	0.22^†^
**hs_CRP(ng/ml)**	3 ± 0.5^a^	4.1 ± 0.7^b^	3.2 ± 0.5^a^	0.01^*^
**PTX-3 (ng/ml)**	0.66 ± 0.09^a^	0.84 ± 0.09^b^	0.68 ± 0.11^a^	0.01^*^
**MMP-9 (ng/ml)**	0.65 ± 0.1	0.74 ± 0.1	0.62 ± 0.1	0.28
**TAC (**μmol**/l)**	227.8 ± 26.9	220.3 ± 16.7	216.6 ± 22.3	0.68
**GPX (U/ml)**	228.3 ± 54.2	220.8 ± 38.8	288 ± 85	0.15
**SOD (U/ml)**	54 ± 20.7^ab^	37.8 ± 14.3^a^	65.9 ± 9.4^b^	0.02^*^
**Steatosis score**	0(0–0.25)^a^	2(1–2)^b^	1(0–1)^a^	< 0.01^†^
**NAS score**	0.5 ± 0.83^a^	3.83 ± 1.16^b^	2 ± 1.09^c^	< 0.001^*^

Values are expressed as mean ± SD for data with normal distribution and median (IQR) for those with non-normal distribution

CCK = Cholecystokinin, hs-CRP = high-sensitivity C-reactive protein, PTX-3 = pentraxin-3, MMP-9 = matrix metalloproteinase-9, TAC = total antioxidant capacity, GPX = glutathione peroxidase, SOD = superoxide dismutase

Different letters show significant difference at *P* < 0.05

† Kruskal–Wallis test

**Fig. 1:**
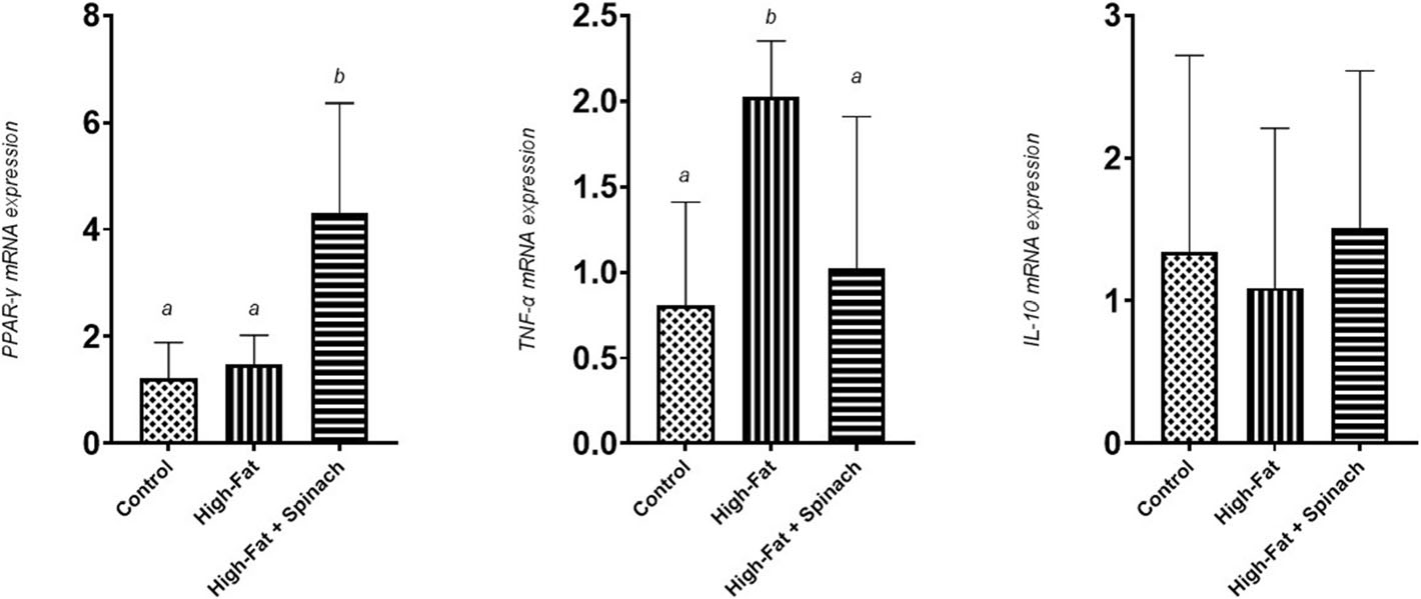
Liver gene expression of PPAR-γ, TNF-α, and IL-10 at the end of the prevention phase. Different letters show significant difference at *P* < 0.05

Receiving HES for seven weeks significantly increased liver gene expression of PPAR-γ when compared to other groups (*P <* 0.01). Liver gene expression of TNF-α was significantly higher in the PF group compared to other groups at the end of the prevention phase (*P <* 0.05). No significant changes were seen in IL-10 levels among the groups (shown in Fig. 1).

### Treatment phase

All of the 24 samples were used for biochemical analysis and the quantification of gene expression in the treatment phase (*n* = 6 in each group). Total weight gain and food intake were significantly higher in rats on the high-fat diet compared to the rats on the normal chow diet (*P <* 0.001). However, receiving HES could not make any significant improvements in body weight and food intake in the treatment phase. Serum levels of hs-CRP and PTX-3 were significantly lower in TFS group compared to TF group at the end of the treatment phase (*P =* 0.02). The rats in TFS group had a higher serum levels of TAC compared to the TF group at the end of the study (*P =* 0.03). Four weeks of supplementation with spinach extract in rats of TCS and TFS groups significantly increased the activity of liver GPX compared to TC and TF groups, respectively (*P =* 0.01). The rats in the high-fat group had higher serum levels of MMP-9 compared to those on the normal chow diet (*P =* 0.02). No statistical differences were observed regarding other markers among other groups (Table 3).

**Table 3:** Weight gain, total food intake, serum inflammation and oxidative stress biomarkers and antioxidant enzyme activity in liver at the end of the treatment phase

	Control (*n* = 6)	Control+ Spinach (*n* = 6)	High-fat (*n* = 6)	High-fat+ Spinach (*n* = 6)	*P* value, ANOVA
Weight gain(g)	−22 ± 22^a^	−22.8 ± 18.4^a^	16.8 ± 7.8^b^	12.3 ± 10.2^b^	< 0.001^*^
Food intake(g)	77.7 ± 5.1^a^	82.1 ± 6.4^a^	111.5 ± 16.8^b^	102.7 ± 7.6^b^	< 0.001^*^
CCK(ng/l)	117.5 ± 48.9	116.4 ± 29.8	127.5 ± 23.1	133.8 ± 17.5	0.75
Hs_CRP(ng/ml)	3.48 ± 0.62^a^	3.68 ± 0.43^a^	4.69 ± 0.96^b^	3.68 ± 0.64^a^	0.02^*^
PTX-3(ng/ml)	0.63 ± 0.14^a^	0.63 ± 0.09^a^	0.86 ± 0.1^b^	0.67 ± 0.17^a^	0.02^*^
MMP-9(ng/ml)	0.5 ± 0.09^a^	0.52 ± 0.19^a^	0.81 ± 0.22^b^	0.64 ± 0.17^ab^	0.02^*^
TAC(μmol/l)	214(181.5–219.2)^ab^	204(199–216.5)^a^	186(180.5–191.2)^b^	209(205.5–212)^a^	0.03^*†^
GPX(U/ml)	233.8 ± 33.5^a^	365.9 ± 115.7^b^	224.6 ± 51.7^a^	336.1 ± 86.1^b^	0.01^*^
SOD(U/ml)	56 ± 25.5	59.9 ± 14.2	44.4 ± 14.6	70.7 ± 16.1	0.12
Steatosis score	0(0–0.25)^a^	0(0–0)^a^	2(1–2)^b^	1(0–1)^a^	0.001^*†^
NAS score	1(0–2)^a^	1(0–2)^a^	4(3–5)^b^	2(0–4)^ab^	0.007^*†^

Values are expressed as mean ± SD for data with normal distribution and median (IQR) for those with non-normal distribution

CCK = Cholecystokinin, Hs_CRP = High-sensitivity C-reactive Protein, PTX-3 = Pentraxin-3, MMP-9 = Matrix metalloproteinase-9, TAC = Total antioxidant capacity, GPX = Glutathione peroxidase, SOD = Superoxide dismutase

Different letters show significantly different at *P* < 0.05

† Kruskal–Wallis test

**Fig. 2:**
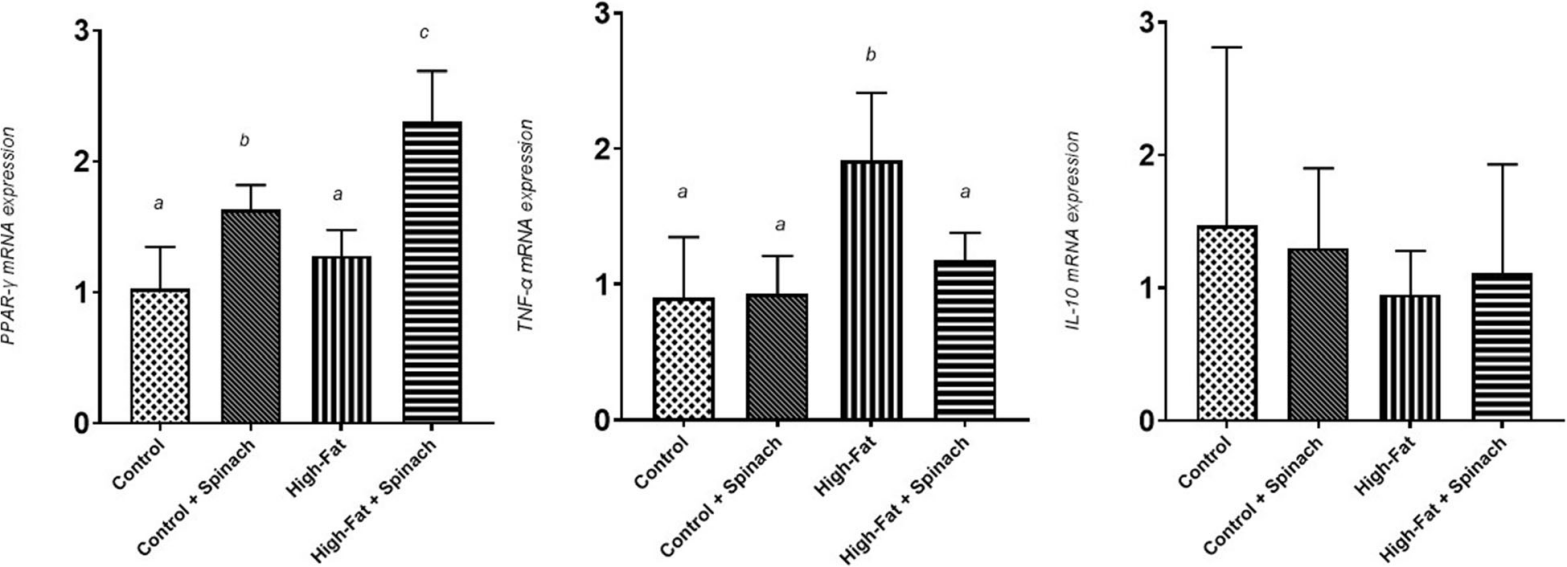
Liver gene expression of PPAR-γ, TNF-α, and IL-10 at the end of the treatment phase. Different letters show significantly different at *P* < 0.05

Supplementation with HES significantly increased liver gene expression of PPAR-γ compared to the control group (*P <* 0.01). Hepatic gene expression of PPAR-γ in TFS group was also higher than in the TCS group at the end of the treatment phase (*P <* 0.01). Liver gene expression of TNF-α was significantly higher in the TF group compared to the TFS group at the end of the treatment phase (*P <* 0.01). No significant changes were seen regarding IL-10 among groups (shown in Fig. 2).

## Discussion

In this experimental study on a NAFLD rat model, we found that compared to the HF diet, HF diet+HES reduced food intake and weight gain in the prevention phase; however, it did not have any impact on these variables in the treatment phase. Furthermore, HES decreased TNF-α gene expression, hs-CRP, and PTX-3; and increased PPAR- γ gene expression and liver SOD activity in the prevention phase. Additionally, HES reduced TNF-α gene expression, hs-CRP, and PTX-3; and increased PPAR- γ gene expression, TAC, and liver GPX activity in the treatment phase.

The intake of HF diet and the subsequent weight gain were higher in rat NAFLD models in both phases of the study, presumably attributable to the greater palatability, softer texture, and higher energy content of this regimen. Adding HES to the diet of rats decreased food intake and led to weight loss in the prevention phase; nonetheless, we were not able to make the same observation in the treatment phase. Previous experimental studies have also shown appetite- and weight-reducing properties of HES supplementation at a dose of 200 mg/kg and 400 mg/kg for 14 days in one study [31], as well as administration of spinach-derived thylakoids for 2 weeks in high-fat fed mice in another [36]. Moreover, in a study of overweight women receiving 5 g/d of thylakoid over a period of 12 weeks, body weight decreased [37]; on the contrary, in a similar study using a dosage of 5.6 g/d administered during eight weeks of intervention, no weight loss was observed [38]. Some of the mechanisms that have been suggested for this appetite-reducing feature of spinach and specifically its active ingredient, thylakoid, include the following: 1) inhibition of lipase/colipase and substrate complex formation [39], 2) reducing the rate of gastric emptying and, in turn, increasing CCK and GLP-1 secretion [17, 18, 37, 40], 3) decreasing ghrelin secretion [41], and 4) stimulation of enterostatin secretion [39]. In the two phases of our study, there was no increase in serum levels of CCK; in contrast to the existing human- and animal-based evidence [17, 31, 39]. Previous studies have measured CCK levels shortly after receiving spinach, while we measured it after 12 h of fasting; therefore, it is deductible that the CCK-promoting effect of spinach is only expected to last for a short period of time and immediately after the meals. The lack of effect of spinach on appetite and weight loss in the treatment phase can be attributed to the shorter duration of intervention and the impairment of appetite-regulatory mechanisms due to the induction of NAFLD using HF diet [42].

Administration of HES improved SOD activity in the prevention phase and promoted GPx enzyme activity in the treatment phase in the liver of the rats. The HES intake also increased serum TAC in the treatment phase. Existing evidence shows that spinach has the highest antioxidant capacity among vegetables [43, 44]. This characteristic has been previously observed in numerous animal and human studies using various types of spinach. In one study, consumption of spinach powder (%5 of diet) for 6 weeks increased SOD activity in liver of the rats [45]. Panda et al. showed that 200 and 400 mg/kg HES for 45 days in rats, as part of a high-fructose diet, enhances the activity of SOD and GPx in heart [46]. In a clinical trial, Bohlooli et al. evaluated the effects of spinach supplementation on antioxidant status and muscle injury in marathon runners. After 14 days of spinach supplementation (1 mg / kg body weight), serum TAC levels were significantly increased compared to the control group [47]. Previous studies have postulated the following mechanisms as possible pathways by which spinach may exert its antioxidant properties: scavenging the radicals of 2,2′-azino-bis(3-ethylbenzothiazoline-6-sulfonic acid) (ABTS), superoxide anion (O_2_), Fe^2+^, peroxyl, and proxy nitrate in in-vitro setting [48, 49]. Evidence suggests that spinach consumption increases serum lutein levels [50, 51]. Lutein can protect the body against radical oxygen species (ROSs) by scavenging the hydroxyl and superoxide radicals, and also inducing antioxidant enzymes (i.e. catalase, SOD, GPx, and glutathione reductase) [52, 53]. Conclusively, spinach may have protective impacts against oxidative stress by enhancing serum levels of lutein and promoting the activity of antioxidant systems of the body; our findings are in line with these observations.

Regarding the possible effect of spinach on inflammatory parameters, we measured the effect of spinach supplementation on some inflammatory cytokines; both in terms of gene expression (IL-10 and TNF-α) and serum levels (hs-CRP and PTX-3). In our study, administration of HES decreased hs-CRP and PTX-3 serum levels and demoted TNF-α gene expression, in both prevention and treatment phases; to our knowledge this observation is unprecedented. Previous to this study, only one clinical trial examined the effect of supplementation with spinach powder (10.4 g / day) on serum CRP levels in which the intake of spinach for 8 weeks had no effect on serum CRP levels in healthy subjects [54]. It is worthy of note that in the present examination, hs-CRP was measured instead of conventional CRP which is more sensitive and is able to detect CRP in smaller quantities [55]. This should also be taken into account that we used spinach extract instead of the foodstuff and it was administered to the rats, not human subjects. This fact may have caused some overestimation as to the efficacy of spinach, thus larger doses of spinach may be needed to create similar effects in human subjects.

Addition of glycolipids extracted from spinach to the in-vitro culture medium of umbilical vein endothelial cells reduced the expression of IL-6 and other lipopolysaccharide (LPS) -induced inflammatory factors by inhibiting phosphorylation and inactivation of the NF-κB pathway [56]. Lutein, found abundantly in spinach, decreased plasma levels of prostaglandin E2, TNF-α, interleukin-1-β, the activity of nitric oxide (iNOS), and cyclooxygenase-2 (COX-2) in the liver; it also inhibited NF-κB signaling pathway that can play an important role in reducing inflammation [57]. Likewise, consumption of spinach antioxidants has been associated with improved LPS-induced inflammation in animal studies [58, 59]. Inflammatory cytokines, especially TNF-α, are the main stimuli of PTX-3 production, an acute phase protein in the body [60, 61]. To sum up, spinach and its compounds are able to inhibit the expression of CRP and PTX-3 by blocking the NF-κB pathway and reducing the production of IL-6, IL-1, and TNF-α [62]. Previous studies have not investigated the effect of spinach on IL-10 as an anti-inflammatory cytokine. Few animal studies have shown that lutein supplementation can increase IL-10 levels [63, 64]. A longer intervention and a higher dose of HES may be needed to increase IL-10 in rat NAFLD models.

The administration of HES in our study did not change serum levels of MMP-9, neither in the prevention nor in the treatment phase. Studies have shown that abnormal accumulation of collagen in the liver and progression of NAFLD to NASH and cirrhosis increase MMP-9 serum levels [21, 22, 65]. We also observed higher serum levels of MMP-9 in the HF diet group compared to the control group; these results are also in line with previous findings. We were not able to find any investigations that had examined the effect of spinach intake on MMP-9 levels; however, a couple of studies have shown favorable effects of quercetin, one of the spinach flavonoids, on reducing MMP-9 levels [66, 67]. In these studies the use of pure quercetin which contains higher amount of quercetin than HES and the small sample size of our study might be the reason as to why we were not able to detect any such impact on MMP-9 levels.

Spinach extract resulted in increased expression of PPAR-γ gene in rat liver in both the prevention and treatment phases. This increase was greater in the prevention phase than in the treatment phase, which appears to be due to the longer period of spinach intake in the prevention phase (7 weeks), compared to the treatment phase (4 weeks). Elvira-Torales LI et al. administered spinach powder to the rats (5% diet) for two weeks; similar to our findings, the expression of PPAR-γ gene in the liver was enhanced compared with the control group [28]. Also, in a study by Stenkula et al. on 30 rats, two weeks of supplementation with spinach thylakoids (33% diet) resulted in increased PPAR-γ gene expression in adipose tissue compared to the control group [36].

Activation of PPAR-γ results in improved insulin sensitivity in peripheral tissues, such as adipose tissue and skeletal muscle; thereby, reducing the transfer of fatty acids to the liver [68–70]. Activation of PPAR-γ reduces insulin resistance by increasing adiponectin levels [71]; adiponectin, in turn, reduces lipid accumulation in the liver and further contributes to the anti-inflammatory effects of PPAR-γ by enhancing PPAR-α activity and fatty acid oxidation [72–74]. PPAR-γ inhibits TGFβ-1 signaling pathway and induces apoptosis that prevents the activation and proliferation of liver stellate cells and their conversion to fibrotic form [75–79]. Previous studies have reported the positive effects of the use of PPAR-γ agonists, e.g. thiazolidinediones, in improving blood glucose, lipids profile, and liver enzymes, and reducing steatosis and inflammation in patients with fatty liver [80, 81]. However, widespread reports of side-effects, such as overweight, heart failure, bone fractures, and bladder cancer have limited the widespread use of thiazolidinediones [82, 83]. Weaker PPAR-γ agonists are naturally present in some plants that partially activate PPAR-γ compared to TZDs. These compounds, known as selective PPAR-γ modulators (SPPARMs) can improve glucose homeostasis without causing the aforementioned side effects [84]. These natural compounds include catechin, quercetin, and kaempferol [85]; all these compounds are present in spinach [86], which can justify the beneficial effects of spinach in improving fatty liver disease.

In this study, a diet consisting of sugar, egg yolk, and butter was used to induce NAFLD in rats, which is a similar dietary pattern most associated with NAFLD pathogenesis in humans. We also used hydro-alcoholic extract that contains both water-soluble and fat-soluble spinach compounds, in contrast to the aqueous or alcoholic extracts. In this study the effects of HES in two phases of prevention and treatment were investigated. In addition, adding the Chow + HES group and comparing it with the Chow group in the treatment phase, allowed us to examine the simultaneous effects of diet modification and spinach intake. Other strengths of the present study are the measurement of the effect of HES on some indices, such as hs-CRP, MMP-9 and PTX-3 for the first time. However, our study also had some limitations: 1) further biochemical analyses and measurements of effective constituents in HES could help us to better interpret our finding; but this was rendered impossible in our study, 2) only a single dose was administered (instead of examining multiple dosages), and 3) lack of dose-related relationships.

Considering the favorable effects of HES on NAFLD indices in the present study, we propose that further clinical investigations be conducted on human subjects with NAFLD.

## Conclusions

In general, the results of this study showed that the administration of HES, when combined with a HF diet, was associated with decreased food intake, weight loss, and increased SOD enzyme activity in the liver of rats. Moreover, hs-CRP, PTX-3, and TNF-α gene expression in the liver were decreased and PPAR-γ gene expression in the liver were increased by spinach administration, both in the prevention and treatment phases. In addition, administration of spinach in the treatment phase was associated with increased serum TAC and increased hepatic GPX enzyme activity. Spinach extract had no effect on CCK, MMP-9, and IL-10 gene expressions.

## Supplementary Information

**Additional file 1.**

**Additional file 2.**

## Data Availability

All data generated or analyzed during this study are included in this published article [and its supplementary information files].
