# Supplementary material for: Administration of hydro-alcoholic extract of spinach improves oxidative stress and inflammation in high-fat diet-induced NAFLD rats
Source: BMC Complement Med Ther. 2021 Sep 3;21:221. doi: 10.1186/s12906-021-03396-x (PMC8418034; doi:10.1186/s12906-021-03396-x)
Supplement: Supplementary file 1 — Additional file 1. [file 12906_2021_3396_MOESM1_ESM.pdf]

name	group	TAC	SOD	GPX	MMP9	Hs_CRP	PTX3	CCK
1	TC	220.00	22.11	286.54	0.608	4.50	0.77	55.40
2	TC	182.00	54.94	260.04	0.488	2.78	0.57	78.18
3	TC	180.00	68.75	217.07	0.382	3.13	0.59	108.36
4	TC	212.00	92.04	232.54	0.620	3.71	0.74	118.61
5	TC	216.00	32.39	208.39	0.408	3.73	0.74	187.50
6	TC	219.00	66.30	198.70	0.542	3.04	0.40	157.34
7	TF	179.00	38.21	164.97	0.836	4.12	0.95	127.16
8	TF	181.00	35.35	208.39	0.657	4.51	0.71	139.69
9	TF	190.00	72.04	180.24	0.511	4.98	0.79	89.00
10	TF	190.00	31.03	303.90	1.120	3.35	0.83	114.06
11	TF	182.00	46.45	260.20	0.991	5.02	0.98	141.96
12	TF	195.00	43.71	229.95	0.794	6.20	0.92	153.35
13	TCS	201.00	52.08	434.15	0.771	3.63	0.46	76.47
14	TCS	193.00	47.82	164.97	0.756	3.58	0.60	122.60
15	TCS	216.00	57.58	441.90	0.386	3.21	0.71	99.25
16	TCS	206.00	53.96	416.78	0.277	4.44	0.69	105.51
17	TCS	218.00	61.03	286.54	0.469	3.87	0.65	131.71
18	TCS	202.00	87.50	451.51	0.505	3.39	0.68	163.04
19	TFS	204.00	72.22	329.95	0.854	3.64	0.64	126.02
20	TFS	206.00	69.47	309.52	0.454	3.62	0.59	103.24
21	TFS	209.00	51.42	451.51	0.466	4.82	0.60	141.96
22	TFS	215.00	69.41	370.22	0.841	2.91	0.94	154.49
23	TFS	211.00	100.00	364.68	0.582	3.80	0.82	139.12
24	TFS	209.00	62.26	191.02	0.696	3.30	0.45	138.55

name	group	steatosis	inflammation	Balloning	total_food	weight	PPAR	IL10	TNF	stea_SCORe	NAS
1	TC	0	1	0	76.10	0.00	1.62	0.41	0.49	0.00	1.00
2	TC	1	0	0	86.70	-14.00	0.83	0.64	0.93	1.00	1.00
3	TC	0	2	0	78.20	-58.00	0.93	3.72	1.28	0.00	2.00
4	TC	0	0	0	71.10	-37.00	0.92	2.38	1.53	0.00	0.00
5	TC	0	0	0	78.70	-20.00	1.18	1.38	0.83	0.00	0.00
6	TC	0	1	1	75.90	-3.00	0.71	0.30	0.35	0.00	2.00
7	TF	2	2	1	103.20	16.00	1.32	0.61	1.50	2.00	5.00
8	TF	1	1	1	120.10	22.00	1.20	0.90	2.12	1.00	3.00
9	TF	2	1	2	111.90	10.00	0.91	1.59	2.32	2.00	5.00
10	TF	2	2	0	86.85	20.00	1.51	1.02	1.43	2.00	4.00
11	TF	1	2	0	109.90	6.00	1.36	0.77	1.49	1.00	3.00
12	TF	2	2	2	137.20	27.00	1.39	0.84	2.62	2.00	6.00
13	TCS	0	0	0	90.60	-10.00	1.81	1.18	0.85	0.00	0.00
14	TCS	0	1	1	77.45	-20.00	1.56	0.96	0.66	0.00	2.00
15	TCS	0	1	1	87.75	2.00	1.59	0.54	1.45	0.00	2.00
16	TCS	0	1	1	80.45	-27.00	1.41	1.09	1.01	0.00	2.00
17	TCS	0	0	0	73.25	-52.00	1.92	2.17	0.73	0.00	0.00
18	TCS	0	0	0	83.35	-30.00	1.53	1.88	0.91	0.00	0.00
19	TFS	1	2	2	105.40	21.00	2.61	1.14	1.16	1.00	5.00
20	TFS	0	0	0	108.60	12.00	2.53	0.77	1.41	0.00	0.00
21	TFS	0	0	0	104.05	-6.00	2.01	0.69	1.38	0.00	0.00
22	TFS	1	1	0	90.85	16.00	1.85	0.81	0.89	1.00	2.00
23	TFS	1	2	1	96.70	9.00	2.10	0.54	1.25	1.00	4.00
24	TFS	1	1	0	111.00	22.00	2.81	2.74	0.99	1.00	2.00
