# Supplementary material for: Administration of hydro-alcoholic extract of spinach improves oxidative stress and inflammation in high-fat diet-induced NAFLD rats
Source: BMC Complement Med Ther. 2021 Sep 3;21:221. doi: 10.1186/s12906-021-03396-x (PMC8418034; doi:10.1186/s12906-021-03396-x)
Supplement: Supplementary file 2 — Additional file 2. [file 12906_2021_3396_MOESM2_ESM.pdf]

name	group	weight	TAC	SOD	GPX	MMP9	Hs_CRP	PTX3	CCK
1	PC	117.00	237.00	58.82	193.24	0.584	3.23	0.63	214.86
2	PC	84.00	213.00	42.46	223.12	0.581	3.66	0.78	104.94
3	PC	66.00	269.00	44.13	164.97	0.576	2.97	0.78	131.71
4	PC	91.00	226.00	51.47	317.32	0.832	3.24	0.59	97.54
5	PC	92.00	234.00	34.44	262.59	0.817	2.02	0.62	178.41
6	PC	114.00	188.00	92.85	209.14	0.511	3.12	0.58	146.52
7	PF	103.00	236.00	62.50	186.83	0.809	3.68	0.90	169.30
8	PF	137.00	224.00	20.80	197.21	0.663	5.41	0.72	100.39
9	PF	176.00	196.00	27.00	243.12	0.634	3.56	0.86	215.43
10	PF	130.00	212.00	40.17	182.10	0.839	3.97	0.73	141.96
11	PF	165.00	241.00	36.60	234.44	0.916	3.65	0.95	219.99
12	PF	146.00	213.00	39.80	281.27	0.603	4.43	0.89	137.98
13	PFS	113.00	205.00	68.75	241.17	0.463	4.12	0.50	128.29
14	PFS	129.00	195.00	50.37	254.40	0.551	2.59	0.80	117.47
15	PFS	111.00	259.00	69.81	269.17	0.702	3.09	0.78	139.12
16	PFS	90.00	218.00	78.94	243.12	0.817	3.11	0.72	131.14
17	PFS	117.00	216.00	62.06	460.20	0.535	3.74	0.70	119.75
18	PFS	137.00	207.00	65.85	260.04	0.674	2.87	0.62	107.22

name	group	steatosis	Inflammation	balloning	total_food	PPAR	IL10	TNF	stea_score	NAS
1	PC	0	1	0	175.05	1.71	4.14	1.57	0.00	1.00
2	PC	0	0	0	149.75	1.32	0.41	0.25	0.00	0.00
3	PC	0	0	0	140.90	0.89	0.87	1.43	0.00	0.00
4	PC	0	0	0	142.60	0.82	0.68	0.62	0.00	0.00
5	PC	0	0	0	142.60	0.35	0.94	0.89	0.00	0.00
6	PC	1	1	0	163.95	2.23	1.02	0.10	1.00	2.00
7	PF	2	1	1	181.10	1.45	0.04	2.10	2.00	4.00
8	PF	1	1	1	156.85	2.33	2.42	2.27	1.00	3.00
9	PF	2	2	2	190.90	1.96	0.19	1.97	2.00	5.00
10	PF	2	2	1	166.15	1.01	2.37	1.39	2.00	5.00
11	PF	1	1	0	177.40	0.98	1.40	2.07	2.00	4.00
12	PF	1	1	0	182.61	1.10	0.12	2.33	1.00	2.00
13	PFS	1	1	1	153.10	2.51	1.25	2.33	1.00	3.00
14	PFS	1	1	1	157.10	2.04	0.38	0.38	1.00	3.00
15	PFS	1	0	0	174.60	7.68	0.74	0.91	1.00	1.00
16	PFS	1	1	1	149.55	4.82	1.76	0.67	1.00	3.00
17	PFS	0	1	0	158.15	3.68	1.43	0.01	0.00	1.00
18	PFS	0	1	0	162.70	5.17	3.53	1.87	0.00	1.00
